# Associations of acute medical care with the transfer and acceptance functions of hospitals in a region in Japan with limited medical resources

**DOI:** 10.1371/journal.pone.0280802

**Published:** 2023-01-23

**Authors:** Takayuki Idaka, Hajime Iwasa, Seiji Yasumura

**Affiliations:** Department of Public Health, Fukushima Medical University School of Medicine, Fukushima, Japan; Bach Mai Hospital, VIET NAM

## Abstract

**Background:**

Japan’s health care system may be providing inpatient care inefficiently with a low number of physicians per hospital bed and a long average length of stay (LOS). The present study examined associations of acute medical care with hospital-level factors, such as the transfer and acceptance rates, and mediation effect of LOS, using medical service fees per day as an outcome measure for the provision of acute medical care in hospitals in a region with limited medical resources.

**Methods:**

To analyze the associations of acute medical care with hospital-level factors, this research used multilevel structural equation modeling (SEM) of a dataset that included 225,203 patients admitted to 99 hospitals in Fukushima, Japan. The characteristics of the patients, medical activities, and hospitals, such as the transfer and acceptance rates, were assumed to have both direct and indirect effects through LOS on medical service fees per day.

**Results:**

The final analysis used data from 165,413 patients discharged or transferred from 79 hospitals. After separating patient-level effects using multilevel SEM, the results revealed that, at the hospital level, the transfer rate had a significant and positive association with increased medical service fees per day, both directly (standardized coefficient [SC] = 0.215) and indirectly (SC = 0.057) through shortened LOS. The number of first hospitalized patients per physician had a significant and positive association with increased medical service fees per day only indirectly through shortened LOS (SC = 0.063). The acceptance rate had a significant and negative association with medical service fees per day only indirectly through prolonged LOS (SC = -0.078).

**Conclusions:**

Hospital-level factors, such as enhanced transfer function, reduced acceptance function, and a large number of patients for treatment of acute episodes per physician, had positive associations with increased medical service fees per day, either directly or indirectly through LOS.

## Introduction

Japan has a universal health insurance coverage system based on the social insurance system that covers all citizens through public medical insurance and allows freedom of choice of medical institutions (free access). Most of the hospitals are privately owned and provide medical services at the official prices set for medical service fees [1]. In the context of the rapid aging of the population and the country’s moderate level of health care expenditure in the percentage of GDP, the health care system provides inpatient care characterized by a low number of physicians and nursing staff per population, a high number of hospitals and hospital beds per population, a low number of physicians and nursing staff per hospital bed, and a long average length of stay (LOS) [2,3].

One of the regions with limited medical resources in Japan is Fukushima Prefecture, located roughly 200 km north of Tokyo with a population of 1,833,152 (21st of 47 prefectures). It has the third largest land area, at 13,784 km^2^, and is among the least populated (40th, with a population density of 133.0 people per km^2^). Fukushima Prefecture’s medical plan, as of 2018, estimates that while the total population will decrease between 2015 and 2025, the percentage of the population aged 65 years and over will increase from 28.8% to 34.5% and that the population will age faster than the national average (26.8% to 30.3%) [4]. The number of hospital beds per 100,000 people in Fukushima Prefecture is 1,345.7, which is higher than the national average (1,229.8), whereas the numbers of hospital physicians per 100,000 people and per 100 hospital beds are 125.8 and 9.4, which are both lower than the national average (159.4 and 13.0, respectively) [5,6]. As of 2017, the average LOS for patients discharged from hospitals is 40.3 days, which is longer than the national average (30.6 days); the average LOS for surgical patients before and after surgery also both tend to be longer than the national average [7]. According to the standardized claims-data ratio after adjusting for age and sex, calculated using data from the National Database of Health Insurance Claims and Specific Health Checkups of Japan established by the Ministry of Health, Labour and Welfare (MHLW), Fukushima Prefecture ranks 44th out of 47 prefectures in terms of hospital discharge support and coordination [8]. In the local system of providing medical care, patients usually receive medical services in the advanced acute and acute phases on their first hospitalization and are then either discharged or transferred to the second hospital that provides care in the post-acute phase. After transfer, patients receive recovery and chronic care at the accepting hospital and are then either discharged or transferred to the third and subsequent hospital that provides the additional care the patient needs. However, if the transfer and acceptance functions of hospitals are not developed, the canonical acute care pathway among hospitals will be obstructed, making it difficult to concentrate medical resources on medical services in the advanced acute and acute phases and shorten LOS. In regions with limited medical resources, the government should promote a division of roles and cooperation of medical functions among hospitals, especially while developing the transfer and acceptance functions of hospitals. Such a setup is critical to establishing an efficient system for providing medical care—by optimizing medical resources and shortening the average LOS.

Lorenzoni et al. [9] conducted an international comparative study that assessed hospital efficiency by measuring LOS and costs of each hospital to elucidate the amount of resources that hospitals in each country use to treat specific conditions and by using multilevel models to distinguish between country- and hospital-level effects. Multilevel models have also been applied for analyses based on patient-level data from hospitals [10–13]. Traditional methods of analysis have often explained individual-level outcomes exclusively in terms of individual-level independent variables. However, when analyzing data corresponding to individuals nested within groups, methods that ignore group membership—by focusing on interindividual variation or individual-level attributes—have the drawback of missing the potential importance of group-level attributes in influencing individual-level outcomes. In addition, if the outcomes for individuals within groups are correlated, then the assumption of independence of observations is violated, resulting in incorrect standard errors and inefficient estimates. The multilevel analysis allows the simultaneous examination of the effects of group- and individual-level predictors—while accounting for the non-independence of observations within groups—and the examination of both interindividual and intergroup variation [14].

Meanwhile, mediation analysis can be used to clarify the extent to which processes of association between independent and dependent variables can be modified, mediated, or confounded by an intermediate variable. A mediation effect occurs when an intermediate variable (the mediator) is responsible for the influence of a given independent variable on a given dependent variable. Research has shown that the characteristics of patients, hospitals, and medical activities have both direct effects on hospitalization costs and indirect effects through LOS [15–18]. However, few studies have combined multilevel and mediation models to analyze the factors and effects on the amount of resources hospitals use to treat patients at the hospital level.

In Japan, it may be possible to provide more cost-effective acute inpatient care (not surgical day case) if cooperating hospitals transfer and accept patients according to their needed care level. Therefore, we assume that differences in hospital care levels and acute care pathways influence the extent of acute inpatient care provided. Specifically, our study aimed to examine the associations between acute medical care and hospital-level factors, such as the transfer and acceptance rates as measures for hospitals’ transfer and acceptance functions, and the mediation effect of LOS. Our research, anchored on the perspective of promoting a division of roles and cooperation among hospitals in regions with limited medical resources, used medical service fees per day as an outcome measure for the provision of acute medical care in hospitals and multilevel structural equation modeling (SEM).

## Methods

### Data source

We used a dataset constructed from patient-level receipt data and diagnosis procedure combination (DPC) data for inpatients between April 1, 2017, and March 31, 2018, as well as hospital-level survey data in Fukushima, with permission from the Fukushima Prefectural Government. The data were collected from 99 hospitals with general beds or beds for long-term care, excluding psychiatric hospitals (excluding one hospital that did not provide data, one hospital that only had beds for long-term nursing care and no inpatient receipt data, and two hospitals that had missing data in some periods) by the Fukushima Prefectural Government. The data were anonymized by the Fukushima Prefectural Government after being matched with each patient between hospitals and did not include patients not covered by insured medical treatment, patients in beds for long-term nursing care, or patients for whom claims were made by a paper receipt. The receipt data included administrative claims information, such as the type, number, date, and cost of each clinical procedure performed and medication administered. The DPC data also included clinical/discharge summaries, such as patient demographics, admission and discharge statuses, and major diagnoses.

### Patient selection

**Fig 1:**
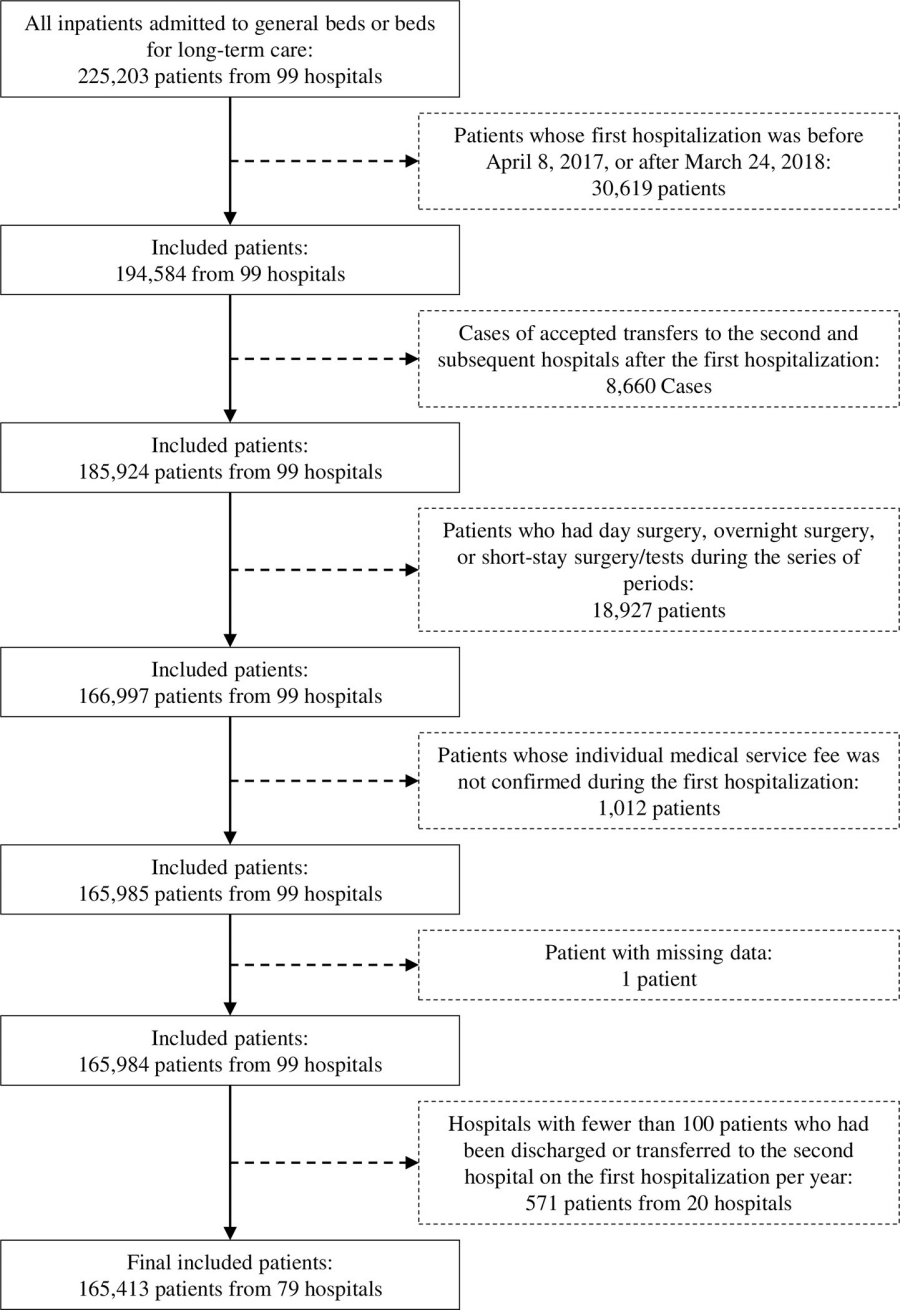
Patient flowchart.

Fig 1 shows the patient selection process. The dataset of all 99 hospital admissions included 225,203 patients admitted to general beds or beds for long-term care, counting each admission as one patient. First, we examined the contents of the receipt and DPC data, and defined cases of transfer/acceptance as those admitted to the next hospital with the same main diagnosis on the day of discharge and within seven days after. Next, for each inpatient, we observed a series of periods, including transfer/acceptance between hospitals, and assumed the earlier admission in the highest staff deployment as the first hospitalization when acute medical care would have started. Based on these results, we selected patients who had been discharged or transferred to the second hospital on the first hospitalization for treatment of acute episodes for our analysis of the provision of acute medical care. Therefore, we excluded patients whose first hospitalization was before April 8, 2017, or after March 24, 2018 (n = 30,619) from the analysis because their status as transferred or accepted during the series of periods was difficult to determine. We also excluded the following types of patients because the focus is on acute medical care: cases of accepted transfers to the second and subsequent hospitals to provide care in the post-acute phase after the first hospitalization (n = 8,660); those who had day surgery, overnight surgery, or short-stay surgery/tests different from typical acute inpatient care during the series of periods including transfer/acceptance between hospitals (n = 18,927); those whose individual medical service fee was not confirmed during the first hospitalization (n = 1,012); and those with missing data (n = 1). Based on the assumption that hospitals with extremely low numbers of patients who had been discharged or transferred to the second hospital on the first hospitalization would have very different hospital functions, mainly dealing with patients after acute care in other hospitals, we selected hospitals with more than 100 such patients per year for analysis and excluded 20 hospitals (n = 571).

### Dependent variable

Our main dependent variables of interest were the medical service fees per day, which were assumed to represent the concentration of medical resources on medical services in the advanced acute and acute phases. First, to focus on the content of the medical care provided to patients in general beds or beds for long-term care in each hospital, we calculated the medical service fees by adding stipulated numbers of points for the individual medical activities provided during hospitalization, with the unit price for one point being JPY 10. The medical service fees per day were calculated by dividing this value by LOS for each patient. These services include medical management, tests, diagnostic imaging, medication, injection, treatment, surgery, anesthesia, radiation therapy, and acute rehabilitation. We excluded rehabilitation after the acute phase because of its focus on acute medical care [9]. Basic fees for inpatient medical care and specific inpatient medical fees were also excluded because they differ depending on the nursing system and other factors, even if the actual medical activities provided were the same or to the same extent. In 2003, the Japanese government introduced the Diagnosis Procedure Combination/Per-Diem Payment System (DPC/PDPS) for reimbursements to acute inpatient care hospitals under the public medical insurance scheme. Medical charges under the DPC/PDPS consist of inclusive and fee-for-service components. The inclusive component covers charges for hospitalization, examinations, and medication, except for expensive procedures, and has a flat rate per-diem fee based on diagnostic categories. However, the medical service fees we used may not necessarily correspond to the medical expenses paid, as they were calculated using only the fee-for-service reimbursement based on the actual medical activities provided to the patient, to account for the amount of medical resources used by each hospital to treat patients. The medical service fees per day were converted from JPY to USD according to the 2017 exchange rate of USD 1 = JPY 112.166 [19].

### Intermediate variable

We considered LOS as an intermediate variable. Medical service fees are typically higher in the first few days after admission, given that more medical care is provided in the acute phase. Therefore, LOS is typically an important factor for medical service fees per day. LOS is also influenced by independent variables, such as the characteristics of patients, medical activities, and hospitals. The medical service fees per day were calculated by dividing the medical service fees by LOS to assess the concentration of medical resources on medical services in the advanced acute and acute phases. However, the average LOS in Japan and Fukushima is long compared to other countries, and the amount of daily medical resources provided to the patient changes over time after admission. Therefore, we included LOS in the model because the impact of LOS is not necessarily properly adjusted by only dividing the medical service fees by LOS.

### Independent variables

#### Patient-level characteristics

We explored the following candidate independent variables: patient age as of admission, sex, death during hospitalization, and individual medical activities provided during hospitalization: surgery, antiarrhythmic agents, antineoplastics, radiation therapy, cardiac catheterization, anticoagulants (oral medicine), emergency medical care, admission to the intensive care unit. Sex was binary coded as “1” if it was a female and “0” if was a male. The death variable was binary coded as “1” if the patient died during hospitalization and “0” otherwise. Each variable of medical activities was binary coded as “1” if it was provided during hospitalization and “0” if not. “Intensive care unit” included admission to ICU, HCU, SCU, PICU, NICU, MFICU, and GCU. “Emergency medical care” included admission to ER, provision of emergency medical care to patients of a greater severity requiring urgent hospitalization, administration of tissue plasminogen activator (tPA) to patients within 4.5 hours after stroke onset, and provision of inpatient care to expectant and nursing mothers requiring urgent hospitalization owing to maternal or fetal condition. Diseases were classified complying with the ICD-10 (2013 version) based on the International Statistical Classification of Diseases and Related Health Problems (ICD) published by the World Health Organization (WHO) to confirm data differences using the data of Fukushima in the Patient Survey by the MHLW.

#### Hospital-level characteristics

We included special functioning hospital/regional medical care support hospital, the number of available beds, number of first hospitalized patients for treatment of acute episodes per physician, transfer rate, and acceptance rate. The special functioning hospital/regional medical care support hospital and number of available beds were based on the hospital-level dataset. The hospital was binary coded as “1” if it was a special functioning hospital/regional medical care support hospital and “0” if not. The number of first hospitalized patients for treatment of acute episodes per physician was calculated by dividing the number of patients who had been discharged or transferred to the second hospital on the first hospitalization based on the patient-level dataset by the number of physicians based on the hospital-level dataset. We calculated the transfer and acceptance rates for each hospital as measures for hospitals’ transfer and acceptance functions, respectively, based on transfer and acceptance cases from the patient-level dataset. The transfer rate was calculated by dividing the number of cases transferred to the second hospital by the total number of cases discharged or transferred to the second hospital on the first hospitalization to focus on acute inpatient care. The acceptance rate was calculated by dividing the number of cases of accepted transfers to the second and subsequent hospitals to provide care in the post-acute phase by the total number of cases, including cases of the first hospitalization and cases of accepted transfers to the second and subsequent hospitals, to focus on recovery and chronic care at the accepting hospital.

### Statistical analyses

We conducted descriptive analyses of patient characteristics, medical activities, medical service fees per day, average LOS in general beds or beds for long-term care, and transfer rate for all inpatients in the dataset and discharged/transferred patients from the first hospitalization, respectively. The results were compared with those of the Patient Survey by the MHLW for patients discharged from hospitals to confirm data differences. We also analyzed the association between the transfer and acceptance rates to confirm their status for each hospital. In addition, we analyzed the intraclass correlation coefficients (ICC) to assess the extent to which observations are not independent of groups for patient-level candidate independent variables and analyzed the within- and between-level correlations for the patient- and hospital-level variables.

We used a multilevel SEM in our analysis, using patient- and hospital-level variables to perform mediation analysis based on the between- and within-models. The medical service fees per day were influenced at the patient level by patient characteristics, medical activities, and LOS; they may also be influenced by not only patient differences but also hospital-level characteristics. Therefore, we included the patient-level variables with ICC values higher than 0.05 as independent variables as they were considered not independent of groups, and we also considered those variables as hospital-level variables in the multilevel model, and further included the hospital-level characteristics as independent variables. LOS was considered as an intermediate variable, and the independent variables were assumed to have both direct effects and indirect effects through LOS on medical service fees per day. Note that while we assumed that differences in hospital care levels and acute care pathways influence the extent of acute inpatient care provided in this study, to examine criteria for assessing the concentration of medical resources on medical services in the advanced acute and acute phases, the treatment and period of care required for inpatients in the acute phase is typically largely determined in part by the disease. Assuming that the simultaneous inclusion in the model would make differences in disease configuration more impactful than differences in hospital care level and that it would be difficult to assess the impact of differences in hospital care level, we decided not to add the classification of diseases to the model to avoid the possibility of overadjustment. Our model parameters were estimated using the robust maximum likelihood method in Mplus. All variables were analyzed as observation variables in this model. Medical service fees per day and LOS were converted into natural logarithms for analysis. The residual variances of the dependent and intermediate variables in the within-model were fixed at 1. Statistical analyses were performed using Mplus (version 8.6, Muthén & Muthén, Los Angeles, CA, USA).

### Ethical considerations

The data collection and analysis were approved (approval number: 2019–143) by the Ethics Committee of Fukushima Medical University. This study was conducted in accordance with the Ethical Guidelines for Epidemiological Research established by the Japanese national government, which stipulate the requirements for protecting patient anonymity. Based on these guidelines, our study satisfied the necessary conditions to waive the need for informed consent, and the ethics committee approved such waiving.

## Results

Our final analysis included 165,413 patients discharged or transferred from 79 hospitals. Table 1 shows the patients’ characteristics. Differences between all inpatients, analyzed discharged/transferred patients, and the results of the Patient Survey by the MHLW were largely due to data exclusions. Data for the analyzed discharged/transferred patients did not include the following: 1) long-term inpatients who had not been discharged for more than one year, 2) patients who had day surgery, overnight surgery, or short-stay surgery/tests, such as diseases of the eye and adnexa and diseases of the digestive system during the series of periods including transfer/acceptance between hospitals, 3) inpatients from psychiatric hospitals, and 4) inpatients who required medical treatment not covered by insurance, such as normal delivery.

**Table 1:** Characteristics of patients and medical activities.

					Analyzed discharged/transferred patients				Patient survey^b^
Characteristic				All inpatients	Number of patients	Medical service fees per day (in USD^a^)	Average length of stay (days)	Transfer rate (%)	Estimated discharged patients (in thousands)
Total (no.)				225,203	165,413	222.8	16.4	4.2	19.4
	Hospitals (no.)			99	79	‡	‡	‡	†
	Number of unique patients (no.)			158,824	124,753	‡	‡	‡	†
Sex (%)									
	Female			48.6	49.3	201.4	17.1	4.4	50.5
	Male			51.3	50.7	245.5	15.8	4.0	49.5
	Unknown			0.1	‡	‡	‡	‡	0.0
Age group (%)									
	0–4 years old			4.7	5.5	142.1	7.3	1.0	6.2
	5–14 years old			2.1	2.4	235.7	6.4	0.9	2.1
	15–24 years old			2.3	2.7	321.5	8.4	1.0	3.1
	25–34 years old			3.8	4.5	251.4	9.3	1.3	5.7
	35–44 years old			4.7	5.3	311.4	10.4	1.5	5.2
	45–54 years old			6.0	6.5	336.3	12.1	2.0	6.2
	55–64 years old			12.0	12.2	319.4	14.3	2.9	11.3
	65–74 years old			21.4	20.7	288.3	15.9	3.8	20.1
	75–84 years old			24.0	22.7	204.4	19.8	5.9	22.7
	85 ≤			18.7	17.5	121.5	24.9	7.6	17.0
	Unknown			0.1	‡	‡	‡	‡	0.0
Classification of diseases^c^ (%)									
	I Certain infectious and parasitic diseases			2.1	2.5	167.3	14.0	3.1	2.1
	II Neoplasms			20.2	21.7	292.6	15.2	2.2	19.1
		Malignant neoplasm		17.4	18.6	279.2	16.2	2.3	16.0
			Malignant neoplasm of stomach	1.9	2.2	272.8	15.9	2.3	2.1
			Malignant neoplasm of colon and rectosigmoid junction and rectum	3.1	3.5	317.6	13.6	1.5	3.1
			Malignant neoplasm of trachea, bronchus, and lung	1.9	2.2	269.0	15.0	2.4	1.5
	III Diseases of the blood and blood-forming organs and certain disorders involving the immune mechanism			0.9	1.0	236.0	17.2	4.4	0.5
	IV Endocrine, nutritional, and metabolic diseases			2.8	3.0	91.6	18.3	3.0	2.6
		Diabetes mellitus		1.2	1.3	79.5	20.0	2.2	1.0
	V Mental and behavioral disorders			0.4	0.3	79.2	13.6	2.4	2.6
	VI Diseases of the nervous system			3.0	2.3	154.2	17.4	4.9	3.1
	VII Diseases of the eye and adnexa			3.5	1.3	333.1	8.9	0.9	3.6
	VIII Diseases of the ear and mastoid process			0.9	1.1	167.4	7.2	0.7	1.0
	IX Diseases of the circulatory system			15.3	15.6	340.6	17.9	7.0	12.9
		Heart diseases (excluding hypertensive)		7.9	8.8	450.0	14.0	2.7	6.7
		Cerebrovascular diseases		5.1	4.3	181.1	27.9	18.1	4.1
	X Diseases of the respiratory system			11.0	12.3	109.1	17.8	3.5	11.9
		Pneumonia		4.6	5.1	91.0	19.7	2.8	5.7
	XI Diseases of the digestive system			12.7	10.6	187.3	13.8	3.1	13.4
	XII Diseases of the skin and subcutaneous tissue			1.0	1.1	98.6	18.6	3.5	1.0
	XIII Diseases of the musculoskeletal system and connective tissue			4.9	5.1	273.2	22.6	4.6	4.6
	XIV Diseases of the genitourinary system			5.0	5.3	163.6	15.0	2.9	4.6
	XV Pregnancy, childbirth, and puerperium			2.7	3.2	180.6	9.6	1.2	4.6
	XVI Certain conditions originating in the perinatal period			1.0	1.1	99.6	12.3	1.8	1.0
	XVII Congenital malformations, deformations, and chromosomal abnormalities			0.5	0.6	557.6	8.8	1.3	0.5
	XVIII Symptoms, signs, and abnormal clinical and laboratory findings, not elsewhere classified			1.5	1.6	90.1	14.4	2.5	1.0
	XIX Injury, poisoning, and certain other consequences of external causes			9.7	9.4	181.4	21.6	10.1	9.3
		Fracture		5.5	5.2	161.2	27.4	14.3	5.7
	XXI Factors influencing health status and contact with health services			0.0	0.0	138.8	3.6	0.0	1.0
	Dental			0.8	1.0	183.5	8.3	0.3	‡
	Unknown			0.2	0.0	196.8	1.8	0.0	‡
Medical activities (%)									
	Surgery			38.2	37.3	378.9	17.9	4.0	36.6
	Antiarrhythmic agents			18.2	20.6	344.9	21.8	5.4	†
	Antineoplastics			8.3	9.9	271.3	16.3	1.4	†
	Radiation therapy			0.8	0.9	250.2	40.6	5.3	†
	Cardiac catheterization			2.2	2.8	502.8	9.4	1.7	†
	Anticoagulants (oral medicine)			18.1	19.1	251.6	24.7	7.4	†
	Emergency medical care			23.0	27.2	184.1	21.3	8.5	†
	Intensive care unit			4.5	5.2	459.9	27.0	6.6	†
Death (%)				6.8	6.5	164.3	28.3	‡	6.7

^a^Exchange rate, USD 1 = JPY 112.166.

^b^Patient Survey 2017 (Ministry of Health, Labour and Welfare). The participants of the survey were patients discharged from hospitals between September 1 and 30, 2017.

^c^Classification of diseases comply with the ICD-10 (2013 version) based on the International Statistical Classification of Diseases and Related Health Problems (ICD) published by the World Health Organization (WHO).

Note. Data not available:

†. Statistics not possible or unsuitable for representation:

‡. Data after rounding estimated figures are lower than 1, which is the minimum digit for representation: 0.0.

**Fig 2:**
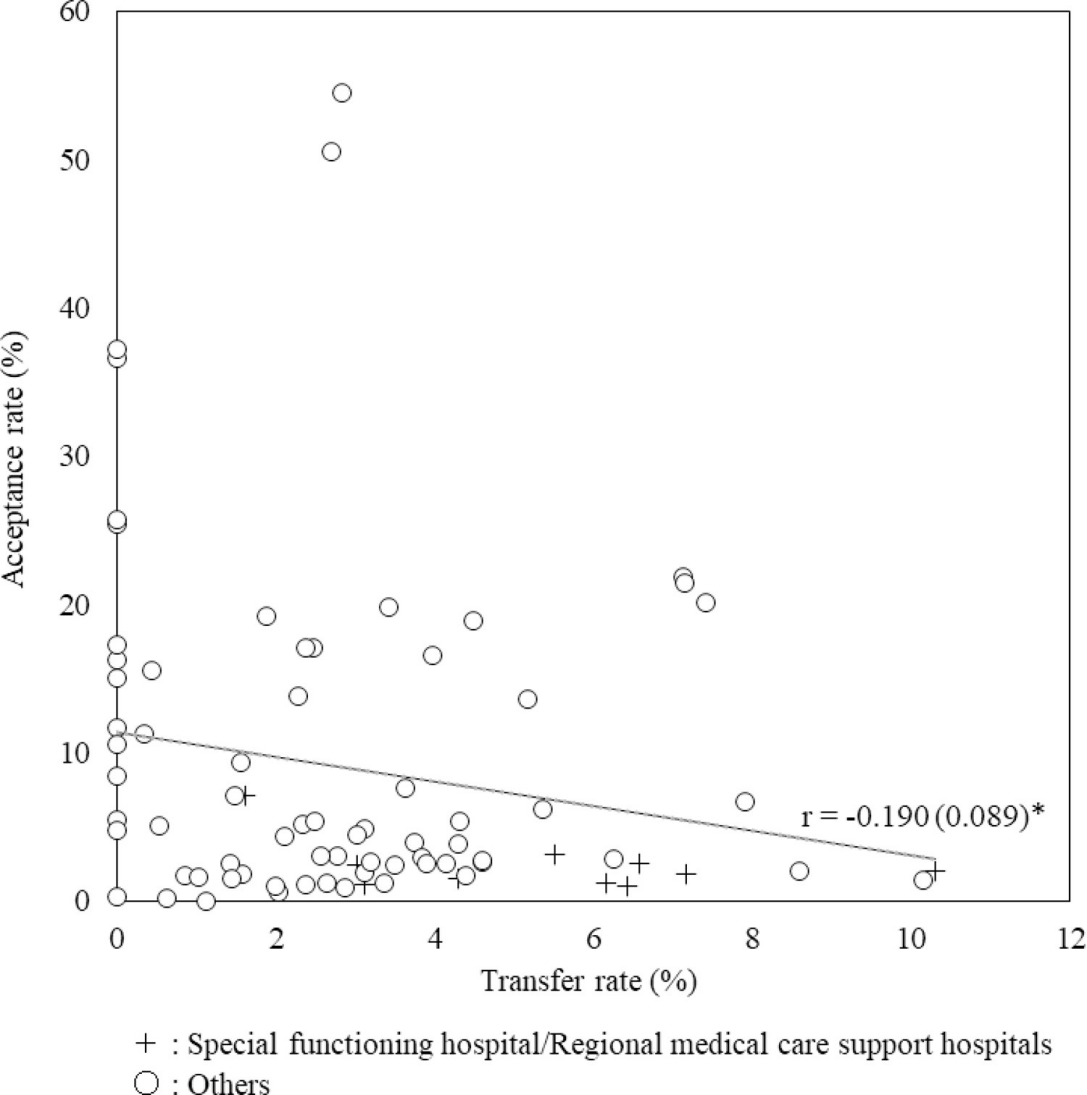
Relation between transfer and acceptance rates for each hospital. Estimate (S.E.), *P < .05, **P < .01, ***P < .001.

The relation between the transfer and acceptance rates for each hospital is shown in Fig 2. There was a very weak correlation between them. The median (minimum to maximum) transfer and acceptance rates in the 10 special functioning hospitals/regional medical care support hospitals were 5.8% (1.6%–10.3%) and 1.9% (1.0%–7.1%), respectively. The median (minimum to maximum) transfer and acceptance rates in the other 69 hospitals were 2.5% (0%–10.1%) and 5.1% (0%–54.5%), respectively.

Table 2 shows the ICC and the correlations for the Within and Between levels. The patient-level variables age, surgery, antineoplastics, emergency medical care, and death were considered not independent of groups, as their ICC values were higher than 0.05.

**Table 2:** Correlations for the within and between levels.

		[1]	[2]	[3]	[4]	[5]	[6]	[7]	[8]	[9]	[10]	[11]	[12]	[13]					
Intraclass correlation coefficient		0.275	0.152	0.227	0.026	0.150	0.044	0.072	0.008	0.036	0.049	0.181	0.034	0.073					
		^***^	^***^	^***^	^***^	^***^	^***^	^**^	^***^	^*^	^***^	^***^	^***^	^***^					
Correlations																			
Variable		[1]	[2]	[3]	[4]	[5]	[6]	[7]	[8]	[9]	[10]	[11]	[12]	[13]	[14]	[15]	[16]	[17]	[18]
Within Level																			
	[1] Medical service fees per day	1.000																	
																	
	[2] Length of stay	-0.214	1.000																
		^***^																	
	[3] Age	0.170	0.272	1.000															
		^***^	^***^																
	[4] Sex (female = 1)	-0.062	0.029	0.024	1.000														
		^***^	^***^																
	[5] Surgery	0.495	0.133	0.017	0.015	1.000													
		^***^	^***^		^*^														
	[6] Antiarrhythmic agents	0.245	0.200	0.117	0.002	0.308	1.000												
		^***^	^***^	^***^		^***^													
	[7] Antineoplastics	0.116	-0.019	0.061	-0.034	-0.129	-0.083	1.000											
		^***^		^***^	^*^	^***^	^***^												
	[8] Radiation therapy	0.008	0.104	0.025	-0.016	-0.039	-0.020	0.133	1.000										
			^***^	^***^	^***^	^***^	^**^	^***^											
	[9] Cardiac catheterization	0.103	-0.092	0.055	-0.056	-0.092	0.049	-0.051	-0.018	1.000									
		^***^	^***^	^***^	^***^	^***^	^***^	^***^	^***^										
	[10] Anticoagulants (oral medicine)	0.088	0.230	0.236	-0.057	0.011	0.241	-0.055	-0.015	0.143	1.000								
		^***^	^***^	^***^	^***^		^***^	^***^	^*^	^***^									
	[11] Emergency medical care	0.022	0.188	0.233	-0.015	-0.140	-0.001	-0.142	-0.032	-0.047	0.113	1.000							
			^***^	^***^		^***^		^***^	^***^	^***^	^***^								
	[12] Intensive care unit	0.146	0.163	0.033	-0.018	0.151	0.211	-0.060	-0.012	-0.006	0.081	0.013	1.000						
		^***^	^***^		^*^	^***^	^***^	^***^	^**^		^***^								
	[13] Death	0.007	0.042	0.160	-0.027	-0.102	-0.007	-0.025	0.021	-0.035	-0.037	0.173	0.023	1.000					
			^**^	^***^	^***^	^***^		^***^	^***^	^***^	^***^	^***^							
Between Level																			
	[1] Medical service fees per day	1.000																	
																	
	[2] Length of stay	-0.586	1.000																
		^***^																	
	[3] Age	-0.406	0.707	1.000															
		^***^	^***^																
	[4] Sex (female = 1)	-0.410	0.278	0.216	1.000														
		^***^																	
	[5] Surgery	0.629	-0.520	-0.540	-0.386	1.000													
		^***^	^***^	^***^	^***^														
	[6] Antiarrhythmic agents	0.440	-0.280	-0.234	-0.371	0.505	1.000												
		^***^	^*^		^***^	^***^													
	[7] Antineoplastics	0.355	-0.192	-0.104	-0.295	0.101	0.144	1.000											
		^***^	^*^		^**^														
	[8] Radiation therapy	0.248	-0.107	-0.148	-0.246	0.158	0.101	0.582	1.000										
		^**^		^*^	^***^			^***^											
	[9] Cardiac catheterization	0.376	-0.222	-0.163	-0.379	0.214	0.471	0.040	0.110	1.000									
		^***^	^**^		^***^	^*^	^***^												
	[10] Anticoagulants (oral medicine)	0.029	0.342	0.433	-0.173	-0.205	0.195	-0.186	-0.115	0.408	1.000								
			^**^	^***^						^**^									
	[11] Emergency medical care	0.324	-0.109	-0.077	-0.133	0.001	0.171	0.074	-0.056	0.161	0.117	1.000							
		^***^								^**^									
	[12] Intensive care unit	0.264	-0.143	-0.241	-0.128	0.293	0.351	0.108	0.303	0.247	0.091	0.104	1.000						
		^***^	^**^	^**^	^*^	^***^	^**^		^***^	^*^									
	[13] Death	-0.486	0.584	0.658	0.450	-0.615	-0.328	-0.130	-0.062	-0.256	0.123	-0.026	-0.161	1.000					
		^***^	^***^	^***^	^**^	^***^	^***^			^***^			^**^						
	[14] Special functioning hospital/Regional medical care support hospital	0.326	-0.164	-0.307	-0.178	0.371	0.393	0.220	0.404	0.224	-0.072	0.186	0.492	-0.252	1.000				
		^***^	^**^	^**^	^*^	^***^	^***^	^*^	^***^			^*^	^**^	^***^					
	[15] Number of available beds	0.350	-0.153	-0.370	-0.305	0.335	0.440	0.322	0.419	0.280	0.039	0.235	0.762	-0.242	0.524	1.000			
		^***^		^**^	^***^	^**^	^***^	^**^	^***^	^*^		^**^	^***^	^***^	^***^				
	[16] Number of first hospitalized patients for treatment of acute episodes per physician	0.284	-0.520	-0.308	0.042	0.214	0.063	0.206	0.058	0.235	-0.168	0.125	0.120	-0.406	0.110	0.098	1.000		
		^**^	^***^											^***^					
	[17] Transfer rate	0.311	-0.122	0.049	-0.208	0.077	0.235	0.002	0.136	0.276	0.363	0.207	0.170	-0.173	0.359	0.178	0.222	1.000	
		^**^								^**^	^**^	^*^			^**^				
	[18] Acceptance rate	-0.505	0.507	0.331	0.091	-0.389	-0.133	-0.063	0.112	-0.072	0.205	-0.322	-0.216	0.530	-0.227	-0.221	-0.487	-0.190	1.000
		^***^	^***^	^***^		^***^					^*^	^***^	^***^	^***^	^***^	^***^	^***^	^*^	

*P < .05,

**P < .01,

***P < .001.

Note. Medical service fees per day and LOS were converted to their natural logarithms.

**Fig 3:**
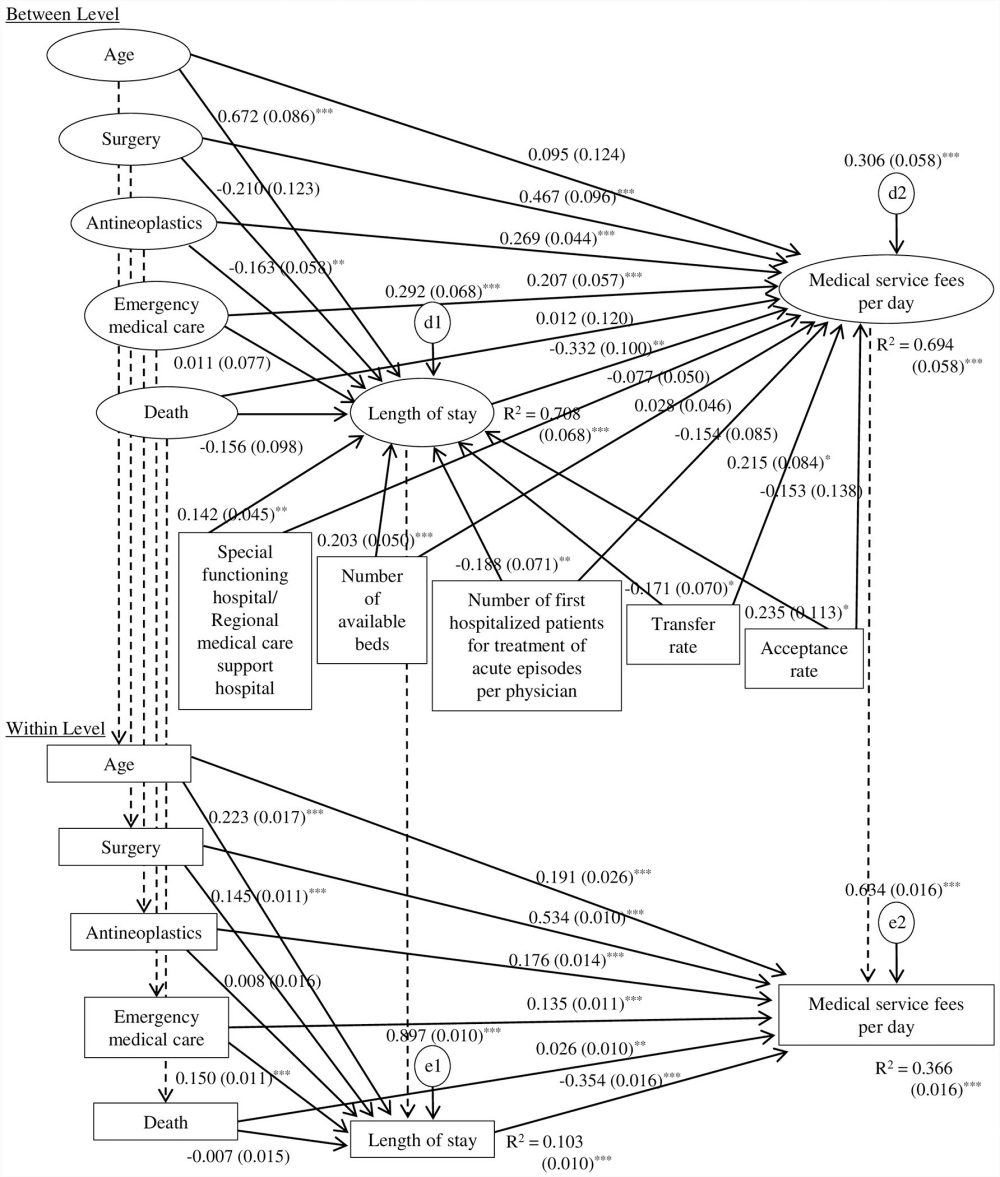
Path showing the relationship between each variable with the standardized coefficients using multilevel structural equation modeling. Estimate (S.E.), *P < .05, **P < .01, ***P < .001. Note. Medical service fees per day and LOS were converted to their natural logarithms.

Fig 3 gives a flow diagram depicting the relationship between each variable and the standardized coefficients of the paths calculated as the results of multilevel SEM using the patient-level variables for which ICC was higher than 0.05 and hospital-level variables. Table 3 presents the direct, indirect, and total effects. The fit indices showed satisfactory model fit (RMSEA = 0.011, CFI = 0.992, SRMR_*B*_ = 0.053, SRMR_*W*_ = 0.068).

**Table 3:** Direct, indirect, and total effects on medical service fees per day with the standardized coefficients using multilevel structural equation modeling.

Variable		Direct effect			Indirect effect			Total effect		
		→ Medical service fees per day (1)			→ LOS → Medical service fees per day (2)			(1) + (2)		
		Estimate	S.E.	P	Estimate	S.E.	P	Estimate	S.E.	P
Within level										
	Length of stay	-0.354	0.016	<0.001				-0.354	0.016	<0.001
	Age	0.191	0.026	<0.001	-0.079	0.009	<0.001	0.112	0.029	<0.001
	Surgery	0.534	0.010	<0.001	-0.051	0.005	<0.001	0.483	0.012	<0.001
	Antineoplastics	0.176	0.014	<0.001	-0.003	0.006	0.619	0.173	0.016	<0.001
	Emergency medical care	0.135	0.011	<0.001	-0.053	0.005	<0.001	0.081	0.011	<0.001
	Death	0.026	0.010	0.007	0.002	0.005	0.655	0.028	0.012	0.016
Between level										
	Length of stay	-0.332	0.100	0.001				-0.332	0.100	0.001
	Age	0.095	0.124	0.443	-0.223	0.072	0.002	-0.128	0.097	0.188
	Surgery	0.467	0.096	<0.001	0.070	0.046	0.131	0.537	0.083	<0.001
	Antineoplastics	0.269	0.044	<0.001	0.054	0.023	0.018	0.323	0.050	<0.001
	Emergency medical care	0.207	0.057	<0.001	-0.004	0.026	0.891	0.204	0.061	0.001
	Death	0.012	0.120	0.923	0.052	0.030	0.086	0.063	0.117	0.586
	Special functioning hospital/Regional medical care support hospital	-0.077	0.050	0.119	-0.047	0.020	0.016	-0.124	0.052	0.018
	Number of available beds	0.028	0.046	0.544	-0.067	0.025	0.008	-0.039	0.045	0.388
	Number of first hospitalized patients for treatment of acute episodes per physician	-0.154	0.085	0.070	0.063	0.031	0.041	-0.091	0.087	0.292
	Transfer rate	0.215	0.084	0.010	0.057	0.027	0.033	0.272	0.084	0.001
	Acceptance rate	-0.153	0.138	0.267	-0.078	0.038	0.042	-0.231	0.133	0.081

Note. Medical service fees per day and LOS were converted to their natural logarithms.

In the between model, LOS had a significant negative association with increased medical service fees per day. The transfer rate and emergency medical care in addition to surgery and antineoplastics had significant direct effects and were positively associated with increased medical service fees per day. As with antineoplastics, the transfer rate had increased total positive effects due to significant indirect positive effects of shortened LOS. The number of first hospitalized patients per physician was significant only due to an indirect positive effect of shortened LOS on medical service fees per day. The negative effects on medical service fees per day were significant only due to indirect effects of prolonged LOS, such as age, acceptance rate, number of available beds, and special functioning hospital/regional medical care support hospital. There was no significant effect of death on medical service fees per day or LOS.

In the within model, LOS had a significant negative association with increased medical service fees per day. All patient characteristics and medical activities had significant direct effects and were positively associated with increased medical service fees per day. Age, emergency medical care, and surgery had reduced total positive effects due to significant indirect negative effects of prolonged LOS.

## Discussion

Our study used a dataset of patients admitted to general beds or beds for long-term care in hospitals in Fukushima, a region with limited medical resources. We identified associations of acute medical care with hospital-level factors, such as the transfer and acceptance rates as measures for hospitals’ transfer and acceptance functions, and the mediation effect of LOS, measuring medical service fees per day as an indicator of the provision of acute medical care in hospitals. The overall results showed that the medical service fees per day, average LOS in general beds or beds for long-term care, and transfer rates were USD 222.8, 16.4, and 4.2%, respectively.

We separated patient-level effects using multilevel SEM. The results showed that at the hospital level, the transfer rate had a significant and positive association with increased medical service fees per day, both directly and indirectly through shortened LOS, similar to the associations of major medical activities. The acceptance rate had a significant and negative association with medical service fees per day only indirectly through prolonged LOS, similar to the association with age. Many previous studies did not consider the importance of a division of roles and cooperation among hospitals in selecting the variables of the characteristics of hospitals for analysis. Our findings highlighted the importance of supporting hospitals that specialize in acute medical care with their enhanced transfer function and reduced acceptance function. This would support them in concentrating their limited medical resources on medical services in the advanced acute and acute phases. Moreover, cooperation should be promoted with medical institutions that provide recovery and chronic care to accept cases transferred from these hospitals in the region.

Our results showed that the number of first hospitalized patients per physician had a significant association with shortened LOS and a significant and positive association with increased medical service fees per day only indirectly. Previous studies have also shown that high-volume surgeons have a shorter LOS compared with low-volume surgeons [20–24]. High-volume surgeons, teaching hospitals, and high-volume hospitals are associated with improved outcomes when providing acute care for a variety of patient conditions and treatments [20,21,24–32]. These reasons are suggested to be related to the skill and experience of individual physicians, composition and experience of the clinical team, appropriate training, effective teamwork, availability of complementary medical and support services, and range of diagnostic and surgical procedures in use. To improve the quality and efficiency of medical care, authorities should consolidate cases for the treatment of acute episodes and promote cooperation among hospitals.

In contrast to the results of previous studies that showed an association between high-volume hospitals and LOS, our results revealed an association between the number of available beds and prolonged LOS. Special functioning hospitals/regional medical care support hospitals also had an association with prolonged LOS. This is consistent with the results of previous studies on teaching hospitals, which suggest that teaching hospitals are thought to treat patients with greater severity than non-teaching hospitals, and that the provision of teaching may require additional time spent on the part of physicians in treating/reviewing each patient so that medical students can learn [9,12,13,21,33]. The number of available beds in our analysis may be attributed to the fact that many high-volume hospitals treat patients of greater severity requiring specialized teams, such as intensive care, pain management, and respiratory care [25,34].

A strength of our study is that the hospital-level characteristics assessed in this analysis are considered representative of the region of Fukushima Prefecture as we used data on inpatients collected from most hospitals with general beds or beds for long-term care in Fukushima. Other data sources need to be used to make the results generalizable to Japan as a whole or to the rest of the world. Notably, data of discharged/transferred patients in the final analysis were generally similar to the results of the statistical survey by the MHLW in the same period and were not considered biased. Therefore, we believe that the results may be applicable to similar prefectures that need to resolve issues related to the division of roles and the cooperation of medical functions among hospitals.

A unique feature of our study is the use of medical service fees per day as the dependent variable. Many previous studies on controlling health care costs have used cost per case rather than cost per day as a measure of relative efficiency [34]. The average LOS in Japan and Fukushima is long, Japan’s per capita medical care expenditure ranks fifth among the G7 countries, and age-adjusted inpatient medical care expenditure per capita in Fukushima also tends to be lower than the national average [35]. For these reasons, it is more important to assess the concentration of medical resources on medical services in the advanced acute and acute phases. In the present study, we used as an indicator the medical service fees per day calculated using only the fee-for-service reimbursement based on the actual medical activities provided to the patient.

### Limitations

Our study has several limitations. First, as our analysis was based on a one-year dataset, we were unable to consider long-term inpatients who had not been discharged for more than one year. Second, owing to the limitations of the dataset, we were unable to include inpatients from psychiatric hospitals and inpatients not covered by insured medical treatment. Further research is needed to elucidate the overall trends in medical services including these patients. Third, the receipt and DPC data we used are originally intended for claiming reimbursements, and as such, they lacked information on the patient’s condition, clinical severity of the disease, purpose of treatment, outcomes, social background, and other details, which might explain the association with the medical service fees per day. Fourth, we defined cases of transfer/acceptance as those admitted to the next hospital with the same main diagnosis on the day of discharge and within seven days after because the data lacked information on outcomes as aforementioned; however, it is possible that patients readmitted with the same main diagnosis were also included. Fifth, the classification of diseases was not added to the model in this study from the perspective of assessing the impact of differences in hospital care levels and acute care pathways on the extent of acute inpatient care provided. Further study is needed to assess this model for each classification of diseases, taking into account the differences among the classification of diseases.

## Conclusions

Hospital-level factors, such as enhanced transfer function, reduced acceptance function, and high volume of patients for treatment of acute episodes per physician, showed positive associations with increased medical service fees per day, either directly or indirectly through LOS. When prefectures formulate and review their medical care plans, they may consider concentrating their limited medical resources on medical services in the advanced acute and acute phases in the future. Particularly, we suggest the incorporation of these three new criteria identified from the results of our study, in addition to the existing criteria of average LOS and amount of each medical activity, to support hospitals that specialize in acute medical care.
